# Sensitivity and Uncertainty Analysis of One-Dimensional Tanaka and Liang-Rogers Shape Memory Alloy Constitutive Models

**DOI:** 10.3390/ma12101687

**Published:** 2019-05-24

**Authors:** A. B. M. Rezaul Islam, Ernur Karadoğan

**Affiliations:** Robotics and Haptics Lab, School of Engineering and Technology, Central Michigan University, Mount Pleasant, MI 48859, USA; islam2a@cmich.edu

**Keywords:** shape memory alloy, Tanaka model, Liang-Rogers model, sensitivity analysis, uncertainty analysis, SMA, shape memory alloy constitutive models

## Abstract

A shape memory alloy (SMA) can remember its original shape and recover from strain due to loading once it is exposed to heat (shape memory effect). SMAs also exhibit elastic response to applied stress above the characteristic temperature at which transformation to austenite is completed (pseudoelasticity or superelasticity). Shape memory effect and pseudoelasticity of SMAs have been addressed by several microscopic thermodynamic and macroscopic phenomenological models using different modeling approaches. The Tanaka and Liang-Rogers models are two of the most widely used macroscopic phenomenological constitutive models for describing SMA behavior. In this paper, we performed sensitivity and uncertainty analysis using Sobol and extended Fourier Amplitude Sensitivity Testing (eFAST) methods for the Tanaka and Liang-Rogers models at different operating temperatures and loading conditions. The stress-dependent and average sensitivity indices have been analyzed and are presented for determining the most influential parameters for these models. The results show that variability is primarily caused by a change in operating temperature and loading conditions. Both models appear to be influenced by the uncertainty in elastic modulus of the material significantly. The analyses presented in this paper aim to provide a better insight for designing applications using SMAs by increasing the understanding of these models’ sensitivity to the input parameters and the cause of output variability due to uncertainty in the same input parameters.

## 1. Introduction

Shape memory alloys (SMAs) have received the attention of researchers due to their unique characteristic behavior and promising potential for various applications. The SMAs, which are classified as smart or intelligent materials, exhibit shape memory effect (SME) and pseudoelasticity (PE) by means of reversible thermoelastic phase transformations between parent phase (austenite) and a product phase (martensite). Shape memory effect is further classified into two types: One-way shape memory effect and two-way shape memory effect. If a SMA material is stressed or deformed, the one-way shape memory effect allows it to come back to its original shape simply by heating. On the other hand, the material exhibiting two-way shape memory effect can be trained to return to another distinct shape by means of cooling. The material must memorize the second effect through a learning process where it stores energy that is freed upon cooling. Shape memory alloy was first discovered by Arne Ölander in 1932 [1] and the term “Shape Memory” was first coined by Vernon in 1941 [2]. In 1962, Buehler and Wang discovered the shape memory effect (SME) [3,4] in a nickel–titanium (NiTi) alloy commonly known as “nitinol”. The necessity and significance of SMAs in engineering applications have been recognized as they are being utilized in automotive, biomedical, and aerospace industries, and in the design of consumer products, mini actuators, micro-electromechanical systems, and robotics. In the automotive industry, SMAs are mainly used as actuators [5,6,7]. The most prominent usage area for SMAs is in the medical field. For instance, in 1975, Andreasen utilized the pseudoelastic property of NiTi alloy to make the first orthodontic implant [8]. Since then, NiTi wires have been extensively utilized in orthodontic procedures [9]. These wires remain in austenitic phase at the temperature of the buccal cavity. Here, pseudoelasticity is exploited for constant force generation after the wires are positioned into the brackets. At the time of insertion, the physician deforms the wire, resulting in a transformation from austenite to martensite. After placement, however, the material transforms into the austenite phase due to increased temperature and, hence, applies constant stress to the contact surfaces while trying to return to its original shape. 

In the orthopedic field, orthopedic staples are used for treating fractures where the stress generated by SMAs is utilized due to constrained heating [10]. The pseudoelastic effect is also exploited in the production of NiTi intramedullary nails [11]. In orthopedics treatments, the SMA properties have been used for physiotherapy of partially atrophied muscles [12]. SMAs are also being used in the vascular field of biomedical applications [13,14,15,16]. In aerospace applications, a Smart Wing program was conducted for optimizing the performance of lifting bodies using active materials including SMAs [17,18,19,20]. Another program named Smart Aircraft and Marine Propulsion System demonstration (SAMPSON) was designed to present the use of active materials in tailoring the inlet geometry and orientation of various propulsion systems [21]. SME actuation was also applied to the adaptable lifting bodies including morphing of the wing structure. In different studies, SMA elements were integrated into the structure of an aircraft [22]. One of the projects aimed to change the configuration of an airfoil from symmetric to cambered due to the actuation of SMA wires [23]. SMAs are used in industry to develop safety devices that can be thermally activated using current interruption mechanisms for the protection of high energy density batteries like lithium ion cells from uncontrollable increase of temperature due to short circuit or overcharging [24]. NiTi SMAs are also used in high-end eyeglass frames. The use of superelastic (or pseudoelastic) SMA components for nosepiece and earpieces provide comfort and resistance to accidental damage. In order to achieve superelasticity over a wide range of temperatures, the eyeglass components are highly cold-worked and then heat treated at low temperature. This way it was possible to impart “work-hardened pseudoelasticity” in them [25,26]. SMAs are also used in micro electro-mechanical devices (MEMS) for optical and electro-optical systems [27]. In robotics, SMAs are mainly being used as actuators [28,29,30].

SMAs transform phase with the application of stress and change of operating temperature. Numerous models have been developed to describe these characteristics. A variety of constitutive laws have been developed including Tanaka and Nagaki [31], Tanaka and Iwasaki [32], Tanaka, Kobayashi and Sato [33], Sato and Tanaka [34], Ivshin and Pence [35], Pence [36], Brinson [37], Brinson and Lammering [38], Boyd and Lagoudas [39], Patoor, Eberhardt and Berveiller [40], Patoor, Eberhardt and Berveiller [41]) and Liang and Rogers [42]. All these models rely on parameters that need to be determined empirically for any given alloy. As a result, the models are subject to experimental uncertainty and random variability in their parameters, which propagate with the application and removal of stress in the material. Consequently, it is necessary to know the most influential set of model parameters. A sensitivity analysis can give a clear idea about the parameters to which a model is most sensitive. It involves testing the robustness of the results of a model or system in the presence of uncertainty. It also provides an understanding of the relationships between input and output variables in a system or model. Karadogan performed a detailed probabilistic evaluation of a one-dimensional Brinson model for its sensitivity to uncertainty in input parameters [43]. In that study, the Brinson model was analyzed to determine which parameters are mostly dominant at different temperature ranges. The output variability was also determined by utilizing a thorough uncertainty analysis of model outputs considering six different cases that included several operating temperatures and loading conditions. However, no work has been done for determining sensitive parameters and uncertainty propagation of the Tanaka and Liang-Rogers models.

In this paper, the Tanaka and Liang-Rogers models were analyzed for propagation of uncertainty to the output stress-strain curve due to the uncertainty present in the input parameters. The sensitivity analysis of these models were also performed for presenting the most influential parameters that contribute to the output variability significantly at various loading/unloading conditions and operating temperatures.

## 2. SMA Constitutive Models

The constitutive models predict the SMA behavior. A constitutive model describes the state of the material in terms of primary variables such as stress, strain and temperature. In this section, we describe the Tanaka and Liang-Rogers SMA models that were used in our analyses. 

### 2.1. Tanaka Model

One of the first SMA constitutive models was developed by Tanaka in 1986. In that study, a thermomechanical framework was constructed that covers the transformation pseudoelasticity and the shape memory effect that is associated with martensitic transformation induced by stress and the reverse transformation. The Clausius–Duhem inequality was utilized to derive the thermomechanical constitutive equations and the kinetics transformations. In this model, it was assumed that unidirectional strain (ϵ), temperature (T) and martensite volume fraction (ξ) are the only state variables. The stress (σ) is calculated as a function of these variables. 

The constitutive equation derived by Tanaka [33] can be written as:

(1) $$\sigma -\sigma_{0}=D\left(\xi \right)\left(\epsilon -\epsilon_{0}\right)+\theta \left(T-T_{0}\right)+\Omega \left(\xi \right)\left(\xi -\xi_{0}\right)$$

Here, D is the elastic modulus of the material, θ is the thermal coefficient of expansion, and Ω is the phase transformation coefficient or the “transformation tensor”. The subscript ‘0’ indicates the initial conditions, i.e., the initial state of the material.

The elastic modulus D is considered a linear function of the martensitic volume fraction ξ and is expressed using the following equation:

(2) $$D\left(\xi \right)=D_{A}+\;\xi \;\left(D_{M}-D_{A}\right)$$

Here $D_{A}$ and $D_{M}$ are termed as elastic constants of the austenite and the martensite. As per Tanaka [33], it was assumed that $D_{A}=D_{M}=D$ (for Cu based SMAs). 

The transformation tensor, Ω, can be represented as:

(3) $$\Omega \;\left(\xi \right)=\;-\epsilon_{L}D\left(\xi \right)$$

where, $\epsilon_{L}$ is the maximum recoverable strain. 

This model uses exponential functions to represent the martensitic fraction. The martensitic fraction is determined during austenite (A) to martensite (M) transformation using the following equation:

(4) $$\xi =1-e^{a_{M}\left(M_{s}-T\right)+b_{M}\sigma }$$

The reverse transformation, i.e., martensite (M) to austenite (A) transformation, has been modeled as:

(5) $$\xi =e^{a_{A\;}\left(A_{s}-T\right)+b_{A}\sigma }$$

Here $A_{s}$, $A_{f}$, $M_{s}$ and $M_{f}$ are known as austenite start temperature, austenite finish temperature, martensite start temperature and martensite finish temperature, respectively. The material constants that have been used here are determined using the following equations:

(6) $$a_{A}=\frac{\ln\left(0.01\right)}{\left(A_{s}-\;A_{f}\right)}\;b_{A}=\frac{a_{A}}{C_{A}}\;a_{M}=\frac{\ln\left(0.01\right)}{\left(M_{s}-\;M_{f}\right)}\;b_{M}=\;\frac{a_{M}}{C_{M}}$$

where, $C_{A}$ and $C_{M}$ are the stress-influence coefficients and are determined from the slope of the critical stress vs. temperature plot [33]. 

For a certain temperature, A→M (austenite to martensite) transformation start stress (A→M_Start) is determined as:

(7) $$\sigma \geq \left(\frac{a_{M}}{b_{M}}\right)\;\left(T-M_{s}\right)$$

And the A→M transformation stop stress (A→M_Stop) can be calculated by:

(8) $$\sigma =\frac{-2\ln10}{b_{M}}+\left(\frac{a_{M}}{b_{M}}\right)\left(T-M_{s}\right)$$

M→A (martensite to austenite) transformation starting stress (M→A_Start) can be determined by using the following equation:

(9) $$\sigma \leq \left(\frac{a_{A}}{b_{A}}\right)\left(T-A_{s}\right)$$

And the M→A transformation stop stress (M→A_Stop) can be calculated by:

(10) $$\sigma =\frac{-2\ln10}{b_{A}}+\left(\frac{a_{A}}{b_{A}}\right)\left(T-A_{s}\right)$$

### 2.2. Liang-Rogers Model

As per the Liang-Rogers model [42], stress, strain, temperature and martensitic fraction provides a complete set of state variables for predicting SMA behavior. The equation that Liang-Rogers use is the rate form of Tanaka’s constitutive equation, i.e., “the unified constitutive equation”. The model can describe the behavior of the SMA materials that have austenite start temperatures greater than martensite start temperatures ($A_{s}\;$>$\;M_{s}$)—there exists another type of SMA material characterized by $A_{s}$ < $M_{s}$. Most commercially available SMA materials belong to the former category; as a result, the Liang-Rogers model considers SMAs with $A_{s}$ > $M_{s}$.

As for the transformation kinetics, Liang-Rogers described the martensite fraction during the austenite to martensite transformation (A→M) as:

(11) $$\xi =\frac{1-\xi_{A}}{2}\cos(a_{M\;}\left(T-M_{f}\right)+\;b_{M}\sigma )+\;\frac{1+\xi_{A}}{2}$$

And for the reverse martensite to austenite transformation (M→A), the equation is

(12) $$\xi =\frac{\xi_{M}}{2}\cos(a_{A\;}\left(T-A_{s}\right)+\;b_{A}\sigma )+\;1$$

Here, $\xi_{A}$ and $\xi_{M}$ are the initial volume fraction for A→M transformation and M→A transformation. 

The material constants are determined using the following equations:

(13) $$a_{A}=\frac{\pi }{\left(A_{f}-\;A_{s}\right)}\;b_{A}=\;\frac{-a_{A}}{C_{A}}\;a_{M}=\frac{\pi }{\left(M_{s}-\;M_{f}\right)}\;b_{M}=\;\frac{-a_{M}}{C_{M}}$$

where, $C_{A}$ and $C_{M}$ indicate the influence of stress on the transition temperatures ($M_{f}$, $M_{s}$, $A_{s}$ and $A_{f}$) and are generally assumed to be the same ($C_{M}=C_{A}$). They are determined from the slope of the stress vs. temperature diagram [42].

The variables for the cosine function in the Liang-Rogers phase transformation equations are limited to the range of 0 to π. Therefore, the martensite to austenite transformation start (M→A_Start) and stop stress (M→A_Stop) range is given by the following equation:

(14) $$C_{A}\left(T-A_{s}\right)-\;\frac{\pi }{\left|b_{A}\right|}\;\leq \;\sigma \;\leq \;C_{A\;}\left(T-A_{s}\right)$$

And the reverse transformation start (A→M_Start) and stop stress (A→M_Stop) range can be derived as:

(15) $$C_{M}\left(T-M_{f}\right)-\;\frac{\pi }{\left|b_{M}\right|}\;\leq \;\sigma \;\leq \;C_{M\;}\left(T-M_{f}\right)$$

## 3. Methods

In order to perform the sensitivity analyses of the Tanaka and Liang-Rogers models, two separate Matlab libraries were developed. The SMA material considered in the analyses was Cu-33.31 Zn-3.17 Sn. The corresponding material properties, which are also referred to as “material constants” in this paper, that were used in the analyses are presented in Table 1. They were determined by Tanaka [33] from the experimental data reported by Pops [44]. The transformation points for the selected alloy were $M_{f}$ = −34 °C, $M_{s}$ = −27 °C $\;A_{s}$ = −25 °C$\;\mathrm{and}\;A_{f}\;$= −14 °C. The critical stress points for the Tanaka model have been calculated using Equations (7) to (10). For the Liang-Rogers model the critical stresses were determined using Equations (14) and (15). Two operating temperatures and maximum loading stresses used in the analyses are presented in Table 2 depending on the critical temperatures of the material. Two different operating temperatures were chosen to observe the models’ behavior in two regions ($T>A_{f}$ and $A_{s}<T<A_{f}$). Additionally, using two different maximum loading stress values allowed us to observe the material behavior when the material completes the martensite transformation upon loading. 

The modeling approach included calculation of the strain values for one-dimensional loading and unloading of the material based on the constitutive equations of each model. The boundary conditions were such that one end of the material was considered as fixed and other end was being pulled by a force during loading until the maximum stress was reached, which was consecutively reduced to zero during unloading. All simulations were performed with the assumption that the material was 100% austenite, i.e., the initial martensite fraction was zero. With every stress increment, martensitic fraction and strain was calculated using the corresponding equations of the Tanaka and Liang-Rogers models.

The simulations were run at two operating temperatures (−10 °C and −22.5 °C) specifically selected to perform our analyses when the material showed the two fundamental characteristics of the SMA’s: pseudoelasticity and the shape memory effect. At −10 °C ($T>A_{f}$), the material showed pseudoelasticity, whereas at −22.5 °C ($A_{s}<T<A_{f}$), the material exhibited the shape memory effect.

In order to observe the effect of maximum loading stress on the sensitivity and uncertainty propagation in both models, the material was loaded up to two different maximum stress values at each aforementioned operating temperature. One of the selected maximum stress values corresponded to the austenite-to-martensite completion stress (A→M_Stop) which is termed as “*σ_max_* = A→M_Stop” in this paper. The second maximum stress was chosen to be greater than A→M_Stop stress, which is termed as “*σ_max_* > A→M_Stop”. Thus, we have considered here four cases for uncertainty and sensitivity analyses at particular temperature and maximum stress (1) −10 °C with 40 MPa (*σ_max_* > A→M_Stop), (2) −10 °C with 36 MPa (*σ_max_* = A→M_Stop), (3) −22.5 °C with 21 MPa (*σ_max_* > A→M_Stop), and (4) −22.5 °C with 17 MPa (*σ_max_* = A→M_Stop).

In order to verify the results of the sensitivity analysis, two variance-based methods for global sensitivity analysis were used: (1) Extended Fourier Amplitude Sensitivity Test (eFAST) and (2) Sobol. The eFAST method is based on Fourier Amplitude Sensitivity Test (FAST) [45,46]. Saltelli et al. [47] proposed the extended FAST (eFAST) to compute the total contribution of each input parameter to the output’s variance. “Total” term here means that the main effect of the parameter as well as the interaction terms involving the parameter are included. The extended FAST method is robust, especially at low sample size, and computationally efficient. The Sobol sensitivity analysis [48] was introduced in 1990s. It is based on decomposition of variance which is achieved by Monte Carlo methods. Sensitivity measures are estimated by Sobol that summarize the model behavior. It calculates the output sensitivity with respect to each parameter individually and the total parameter sensitivity that includes interactions. 

The input parameter values have been extracted from the constitutive equations and the phase transformation equations. The constitutive equation (Equation (1)) considers D, θ and Ω as input parameters since their values have significance on the resulting strain. The martensitic fraction exponential equation during austenite to martensite transformation (Equation (4)) depends on constants $a_{M}$, $b_{M},$ $M_{s}$ and T. On the other hand, martensitic fraction equation (Equation (5)) during reverse transformation (i.e., martensite to austenite transformation) depends on constants $a_{A}$, $b_{A}$, $A_{s}$ and T. Additionally, $a_{A\;}$depends on $A_{s}$ and$\;A_{f}$, while $a_{M}$ depends on $M_{s}$ and $M_{f}$ (Equation (6)). Consequently, eight input parameters have been determined for this study: operating temperature (T), the material elastic modulus (D), phase transformation coefficient (Ω), thermal coefficient of expansion (θ), martensite start temperature ($M_{s}$), martensite finish temperature ($M_{f}$), austenite start temperature ($A_{s}$) and austenite finish temperature ($A_{f}$). These parameters have been considered to have a normal distribution probability density function with coefficient of variation (COV) of 0.01 for all parameters. The nature of SMA materials dictates that the martensite finish temperature be less than the martensite start temperature, and that the austenite start temperature be less than the austenite finish temperature. The normal distributions for these parameters with a higher COV value than 0.01 caused overlaps during the sampling stage of the analysis. These overlaps violated the physical nature of the material and, therefore, constitutive equations failed to explain the related phenomena under those circumstances. Therefore, a COV of 0.01 has been considered to prevent these issues. The probability distribution of the input parameters are provided in Table 3. 

**Figure 1:**
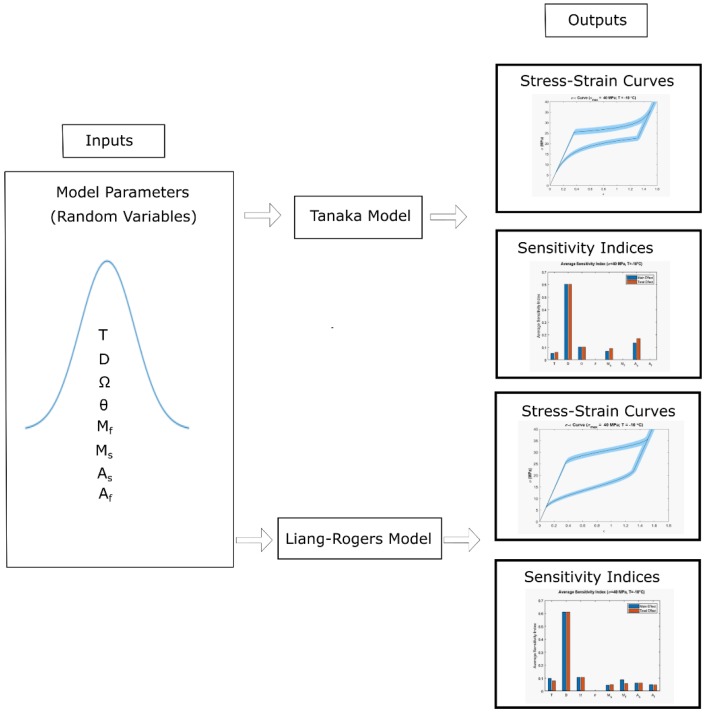
Uncertainty in the stress-strain curves and sensitivity indices as outputs for Tanaka and Liang-Rogers models using eight model input parameters.

The table shows the deterministic values of the input parameters as the mean value of the normal distribution with corresponding standard deviations. The material was stressed from zero to a maximum stress and then the stress was reduced back to zero. The stress increment was selected to be 0.1 MPa at all times. At every stress increment and decrement, the corresponding strain values for each model were calculated. With these values, stress vs. strain output curves were obtained for both the Tanaka and Liang-Rogers models. The propagation of uncertainty due to the variation in the input parameters during loading and unloading of the material were reflected in the corresponding stress-strain curve for both models. A total of eight normally distributed parameters were used as inputs and corresponding stress-strain curves and sensitivity indices charts were generated as outputs for both the Tanaka and Liang-Rogers models (Figure 1).

## 4. Results

**Figure 2:**
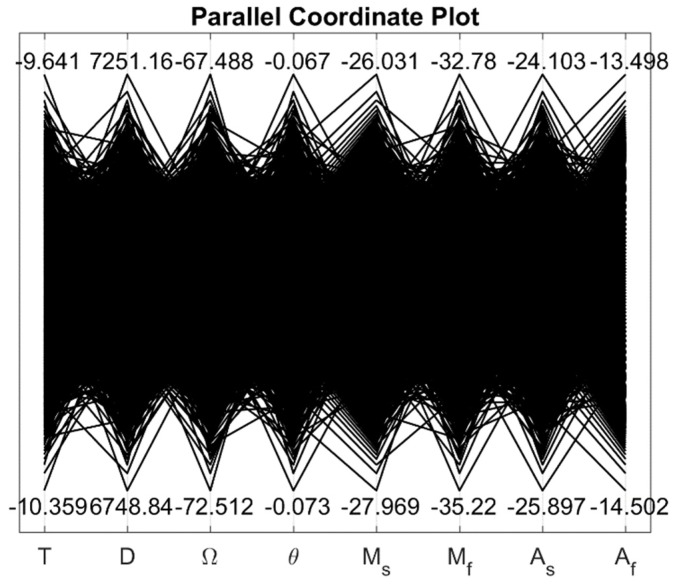
Parallel coordinate plot showing the upper and lower limits of the input parameters (the limits are shown for the temperature −10 °C).

With the uncertainty present in the input parameters, output strain showed significant variability at simulated stress and temperature values. The parallel coordinate plot in Figure 2 shows the upper and lower limits of the normally distributed input parameters. The maximum variability for the Tanaka model and the Liang-Rogers model are presented in Table 4 at different operating temperatures.

### 4.1. Uncertainty Analysis

The uncertainty present in the input parameters propagated to the output stress-strain curves. As a general observation, the model output varied with temperature and loading conditions. In this section, uncertainty analysis results of the Tanaka and Liang-Rogers models are presented. 

#### 4.1.1. Uncertainty Analysis for Tanaka Model 

**Figure 3:**
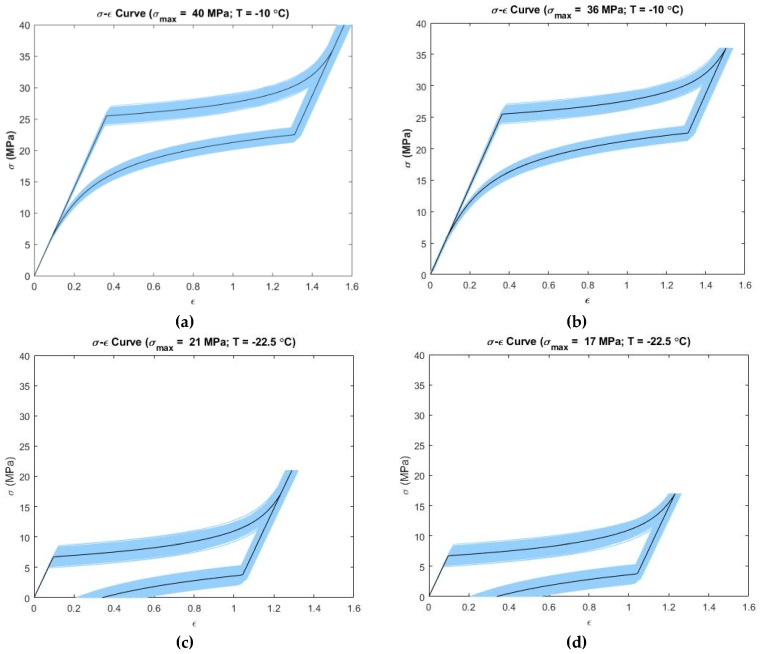
Confidence intervals (5–95 percentile) at simulated temperatures and maximum loading stress for the Tanaka model (the deterministic curve is shown in dark color; the deformation size is shown in the form of strain (ϵ) on the x-axis): (**a**)$\;T=-10\;{}^{\circ}C,{\;\sigma }_{max}=40\;$ MPa; (**b**)$\;T=-10\;{}^{\circ}C,{\;\sigma }_{max}=36$ MPa; (**c**) $T=-22.5\;{}^{\circ}C,{\;\sigma }_{max}=21\;$ MPa; (**d**)$\;T=-22.5\;{}^{\circ}C,{\;\sigma }_{max}=17$ MPa.

Figure 3 presents the propagation of uncertainty to the output for the Tanaka model at simulated operating temperature and loading conditions. From Figure 3a which is termed as *σ_max_* > A→M_Stop and Figure 3b which is termed as *σ_max_* = A→M_Stop, it is observed that the loading portion of the curves showed very low variability at the initial linear region. In the nonlinear loading portion, the variability increased. On the other hand, in unloading linear portion, the variability decreased. The variability increased again in the unloading nonlinear region of the curves. Maximum variability was 56–137% at −10 °C for both Figure 3a,b. It is observed from Figure 3c,d that the initial linear loading region showed low variability in strain, which increased in the nonlinear loading portion. In the unloading linear region, this variability decreased but it again increased in the nonlinear unloading region. Maximum variability was 82–583% at −22.5 °C for both Figure 3c,d. Figure 3a,b shows uncertainty propagation in “pseudoelastic” behavior of SMAs and Figure 3c,d shows uncertainty propagation in “shape memory effect” behavior of SMAs as per the Tanaka model. 

#### 4.1.2. Uncertainty Analysis for Liang-Rogers Model

**Figure 4:**
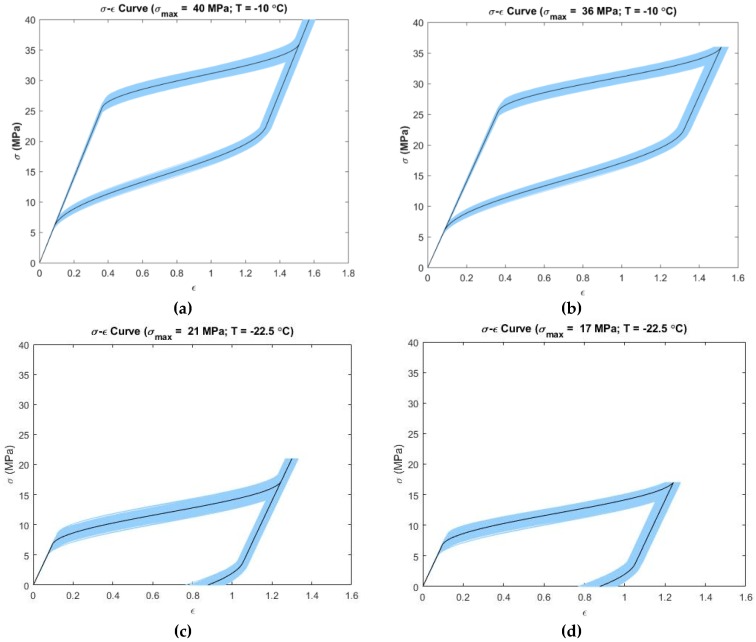
Confidence intervals (5–95 percentile) at simulated temperatures and maximum loading stress for the Liang and Rogers model (the deterministic curve is shown in dark color; the deformation size is shown in the form of strain (ϵ) on the x-axis): (**a**)$\;T=-10\;{}^{\circ}C,{\;\sigma }_{max}=40\;$ MPa; (**b**)$\;T=-10\;{}^{\circ}C,{\;\sigma }_{max}=36$ MPa; (**c**) $T=-22.5\;{}^{\circ}C,{\;\sigma }_{max}=21$ MPa; (**d**)$\;T=-22.5\;{}^{\circ}C,{\;\sigma }_{max}=17$ MPa.

Figure 4 presents the propagation of uncertainty to the output for the Liang-Rogers model at simulated operating temperature and loading conditions. In Figure 4a,b it is observed that the linear loading region showed low variability. The variability increased in the nonlinear loading region. In the linear unloading region, the variability decreased. The variability increased again in the beginning of nonlinear unloading region and tended to decrease towards the end of unloading. Maximum variability was 22–28% at −10 °C for both Figure 4a,b. At the temperature −22.5 °C, as per Figure 4c,d, the initial linear loading region showed low variability in strain. Then, it increased in the nonlinear loading portion. In the unloading linear region, this variability decreased but it again increased in the nonlinear unloading region. Maximum variability was 48–105% for both Figure 4c,d. Figure 4a,b show uncertainty propagation in “pseudoelastic” behavior of SMAs and Figure 4c,d show uncertainty propagation in “shape memory effect” behavior of SMAs according to the Liang-Rogers model. 

The above statements can be verified utilizing the maximum variability data shown in Table 4. As the Tanaka model uses an exponential function, there were sharp increases or decreases in strain values during loading and unloading. With the uncertainty present in the input parameters, the resultant variability is higher for the Tanaka model. The Liang-Rogers model uses a cosine function for which the resultant stress-strain curve is convex shaped. The strain values did not increase sharply as like Tanaka. As a result, with the uncertainty present in the input parameters, the variability was lower in the Liang-Rogers model. It was prominent that for both the Tanaka model and the Liang-Rogers model, the maximum variability was higher for −22.5 °C than −10 °C in all conditions. The maximum variability in a certain temperature for the Tanaka model and the Liang-Rogers model was the same for both *σ_max_* > A→M_Stop and *σ_max_* = A→M_Stop.

### 4.2. Sensitivity Analysis

**Figure 5:**
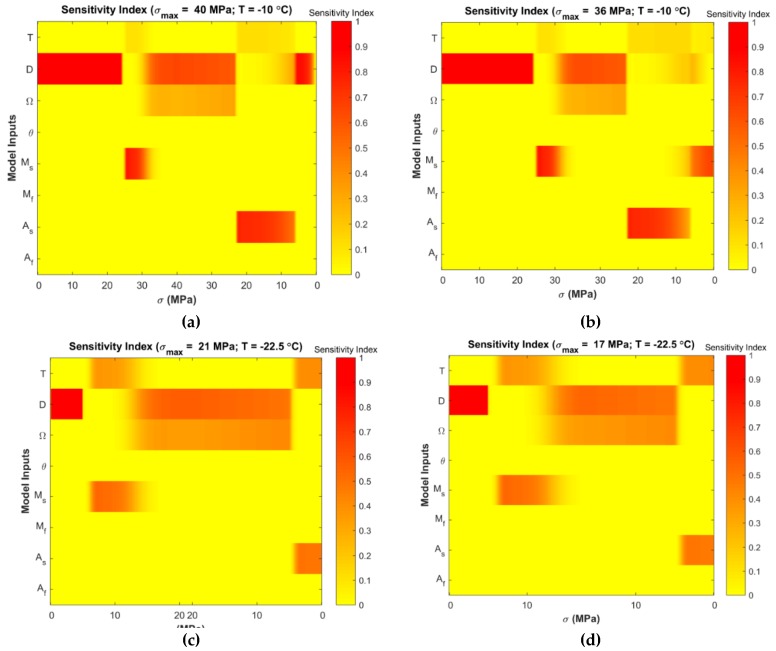
Extended Fourier Amplitude Sensitivity Test (eFAST) stress-dependent sensitivity index distribution at simulated temperatures for the Tanaka model (the corresponding stress values during loading and unloading are shown in horizontal axis and inputs are shown in vertical axis): (**a**)$\;T=-10\;{}^{\circ}C,\;\sigma_{max}=40\;$MPa; (**b**)$\;T=-10\;{}^{\circ}C,\;\sigma_{max}=36$ MPa; (**c**) $T=-22.5\;{}^{\circ}C,\;\sigma_{max}=21$ MPa; (**d**)$\;T=-22.5\;{}^{\circ}C,\;\sigma_{max}=17$ MPa.

Variance-based global sensitivity analyses were performed to determine the most influential parameters of the Tanaka and Liang-Rogers models. Figure 5 and Figure 8 show the stress-dependent sensitivity index distributions at simulated temperatures for the Tanaka and Liang-Rogers models, respectively. It was observed that the sensitivity index varied with temperature and loading region as expected. Main and total sensitivity indices were also calculated for each parameter at 0.1 MPa stress increment. In the next subsections, Tanaka and Liang-Rogers sensitivity analysis results are presented individually. 

#### 4.2.1. Sensitivity Analysis for Tanaka Model 

Figure 5a,b show that the elastic modulus D was dominant during the linear loading region. Phase transformation coefficient Ω showed contribution during austenite to martensite transformation. Austenite start temperature $A_{s}$ showed some significance during nonlinear unloading region where martensite was converted to austenite. Figure 5c,d show the significance of elastic modulus in the initial loading portion. During the austenite to martensite phase transformation region and then in the unloading region, elastic modulus and phase transformation coefficient played significant roles as evidenced by their sensitivity indices. 

**Figure 6:**
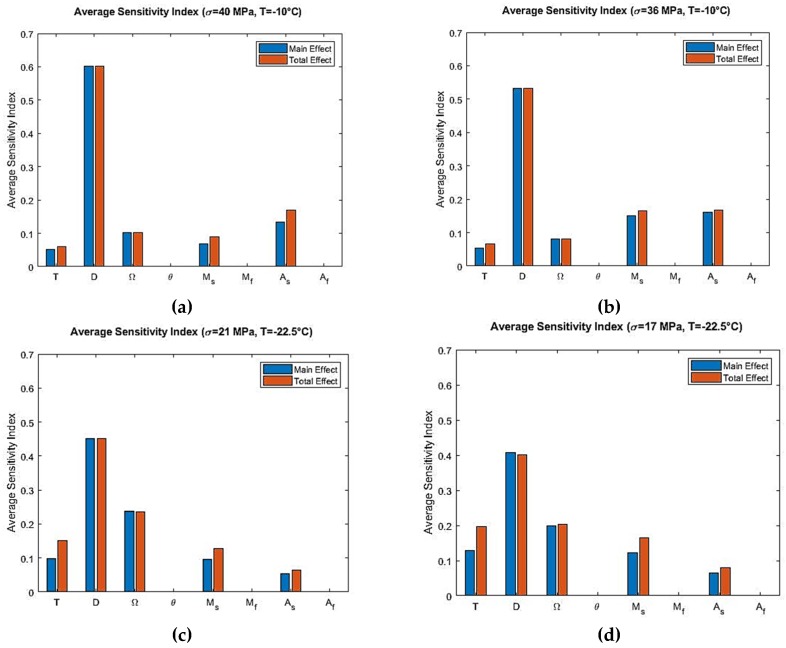
Sobol average sensitivity indices at simulated temperatures and maximum loading stress for the Tanaka model: (**a**)$\;T=-10\;{}^{\circ}C,\;\sigma_{max}=40\;$MPa; (**b**)$\;T=-10\;{}^{\circ}C,\;\sigma_{max}=36$ MPa; (**c**) $T=-22.5\;{}^{\circ}C,\;\sigma_{max}=21$ MPa; (**d**)$\;T=-22.5\;{}^{\circ}C,\;\sigma_{max}=17$ MPa.

**Figure 7:**
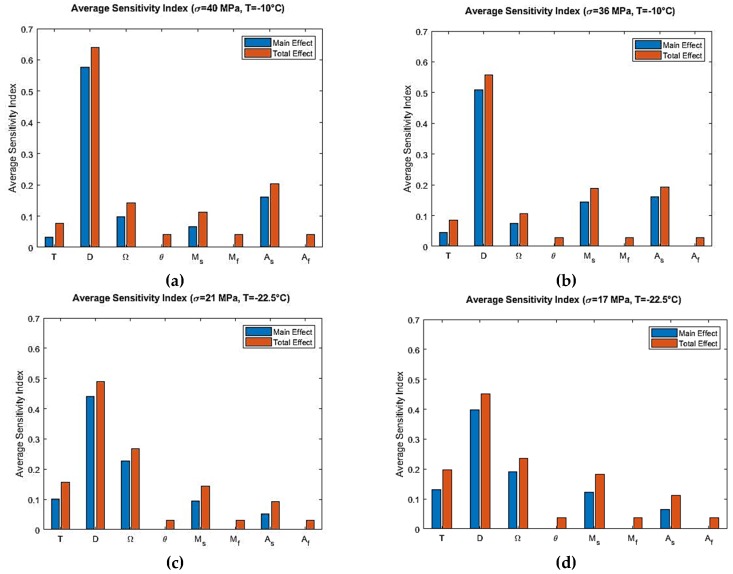
eFAST average sensitivity indices at simulated temperatures and maximum loading stress for the Tanaka model: (**a**)$\;T=-10\;{}^{\circ}C,\;\sigma_{max}=40\;$MPa; (**b**)$\;T=-10\;{}^{\circ}C,\;\sigma_{max}=36$ MPa; (**c**) $T=-22.5\;{}^{\circ}C,\;\sigma_{max}=21$ MPa; (**d**)$\;T=-22.5\;{}^{\circ}C,\;\sigma_{max}=17$ MPa.

From the Sobol sensitivity analysis, the main effect and the total effect sensitivity indices were obtained and the average sensitivity indices were calculated with the resulting data. Sobol average sensitivity index vs. input parameters are presented in Figure 6 for the Tanaka model. It was observed that, apart from the main effect, there were no significant interactions between the parameters. Hence, the total effect was in close agreement with the main effect. The parameters θ, $M_{f}$ and $A_{f}$ had no effect in the output variability as per the Sobol analysis for the Tanaka model. In order to verify these results for the Tanaka model, the sensitivity analysis were repeated by using the eFAST method. The resulting average sensitivity indices are presented in Figure 7.

#### 4.2.2. Sensitivity Analysis for Liang-Rogers Model 

**Figure 8:**
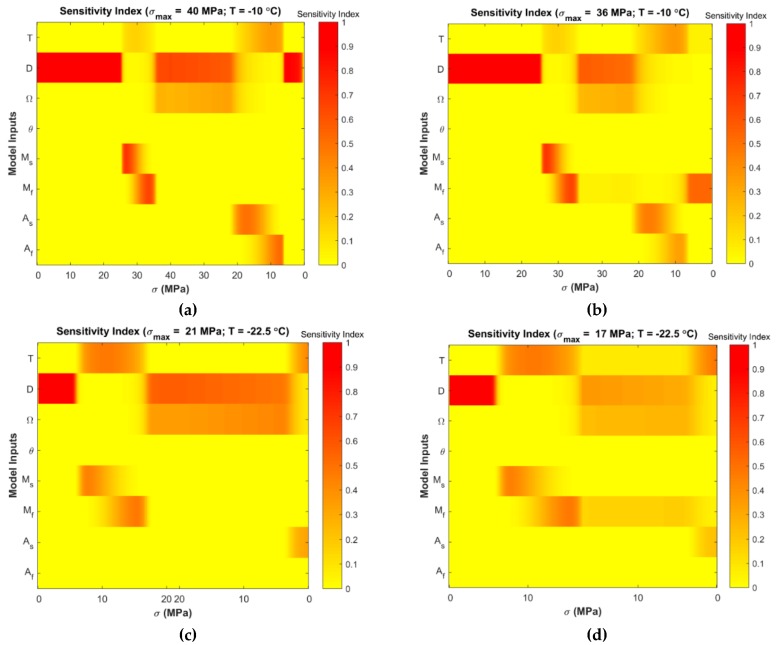
eFAST stress-dependent sensitivity index distribution at simulated temperatures for the Liang-Rogers model (the corresponding stress values during loading and unloading are shown in horizontal axis and inputs are shown in vertical axis): (**a**) $T=-10\;{}^{\circ}C,\;\sigma_{max}=40\;$MPa; (**b**)$\;T=-10\;{}^{\circ}C,\;\sigma_{max}=36$ MPa; (**c**) $T=-22.5\;{}^{\circ}C,\;\sigma_{max}=21$ MPa; (**d**)$\;T=-22.5\;{}^{\circ}C,\;\sigma_{max}=17$ MPa.

Figure 8a,b show that the elastic modulus (D) remained influential in the initial loading region for the Liang-Rogers model. Martensite finish temperature $M_{f}$ was also a parameter for which the model was sensitive during austenite to martensite transformation region and at the ending portion of unloading. Figure 8c,d show high sensitivity index for elastic modulus in the loading region. Then, the phase transformation coefficient Ω became dominant as shown in Figure 8c. Figure 8d reveals that the operating temperature T and martensite finish temperature $M_{f}$ were influential parameters.

**Figure 9:**
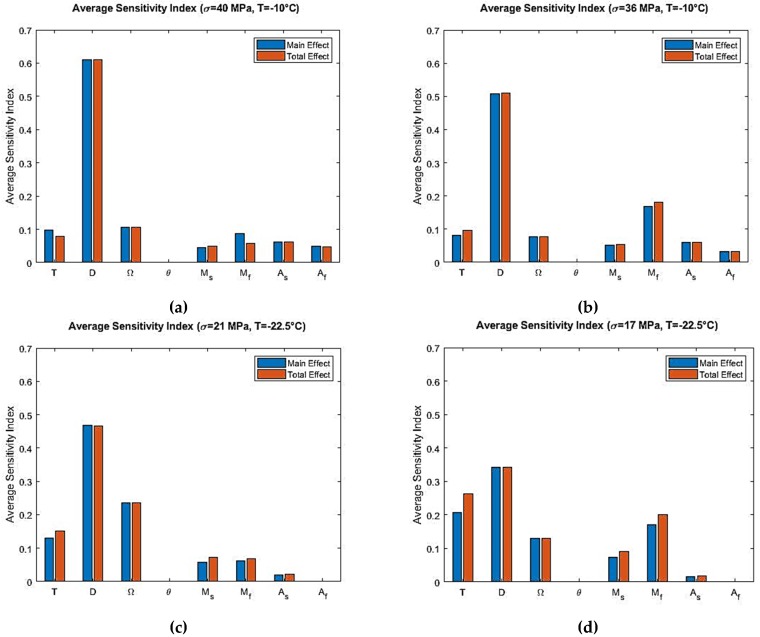
Sobol average sensitivity indices at simulated temperatures and maximum loading stress for the Liang-Rogers model: (**a**)$\;T=-10\;{}^{\circ}C,\;\sigma_{max}=40$ MPa; (**b**)$\;T=-10\;{}^{\circ}C,\;\sigma_{max}=36$ MPa; (**c**) $T=-22.5\;{}^{\circ}C,\;\sigma_{max}=21$ MPa; (**d**)$\;T=-22.5\;{}^{\circ}C,\;\sigma_{max}=17$ MPa.

Main effect and the total effect sensitivity indices were obtained using Sobol sensitivity analysis for the Liang-Rogers model, and the average sensitivity indices were calculated with the resulting data. Sobol average sensitivity index versus input parameters are presented in Figure 9 for the Liang-Rogers model. θ had no effect in all simulated conditions. Figure 9c,d present that $A_{f}$ had also no effect for model sensitivity. $M_{f}$ showed contribution for the conditions presented in Figure 9b,d. 

**Figure 10:**
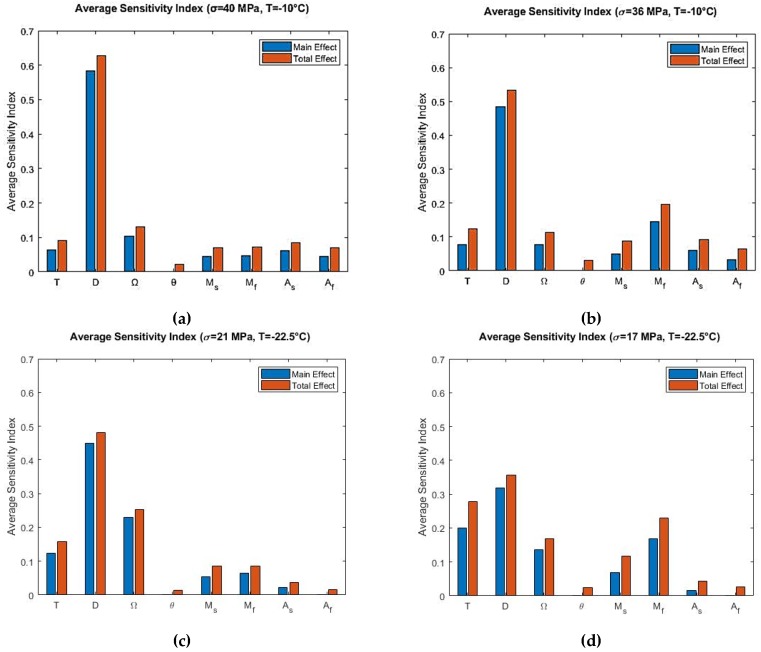
eFAST average sensitivity indices at simulated temperatures and maximum loading stress for the Liang-Rogers model: (**a**) $T=-10\;{}^{\circ}C,\;\sigma_{max}=40$ MPa; (**b**)$\;T=-10\;{}^{\circ}C,\;\sigma_{max}=36$ MPa; (**c**) $T=-22.5\;{}^{\circ}C,\;\sigma_{max}=21$ MPa; (**d**)$\;T=-22.5\;{}^{\circ}C,\;\sigma_{max}=17$ MPa.

From these analyses, it is observed that the most significant parameter was the elastic modulus, D, which contributes to the output variation during the initial loading region, at the end of phase transformation from austenite to martensite and in the beginning and mid-region of unloading. In order to verify these results for the Liang-Rogers model, the sensitivity analysis were repeated by using the eFAST method. The resulting average sensitivity indices are presented in Figure 10.

## 5. Discussion

In this paper, Tanaka and Liang-Rogers shape memory alloy constitutive models were analyzed for sensitivity to input parameters and uncertainty propagation in the output stress-strain curves. We employed a probabilistic evaluation approach that is operated by assigning probability distributions to the input parameters and provides insight into the most influential set of parameters for a given model. The methodology and results presented in this paper can benefit the real life experimentation or applications of SMAs with these models as it reveals the most influential parameters for the considered models. Without proper understanding of these simulations and results, real-life applications may have performance discrepancies. For example, when SMAs are used as dental braces, the recovery effect of the SMAs is utilized for aligning and straightening the teeth. The body temperature causes the braces to put constant recovery stress on the teeth. The design of these braces is done following an SMA model. The deterministic parameters which are the model inputs may effectively provide an expected output. When uncertainty is present in the input parameters, however, the resulting output can go outbound and eventually fail to align and straighten the teeth. In the next paragraphs, the results obtained from the analysis are discussed for simulated cases and recommendations are provided for making use of the Tanaka and Liang-Rogers models in SMA applications. 

As per the Tanaka model, at temperature −10 °C with maximum stress of 40 MPa, the linear loading region shows very low variability. In this region, the material is initially at 100% austenite phase, i.e., no phase transformation is present in this region and the martensitic fraction is always zero. Therefore, T, θ and Ω are not utilized in the constitutive equations. Therefore, low variability is present in the output strain. 

The most variability is observed in the phase transformation regions. These are the regions during transformation of austenite to martensite and vice versa. In these regions, martensite fraction comes into effect involving the exponential function in Equations (4) and (5). These equations involve the parameters $a_{M}$, $M_{s}$, T, $b_{M}$, $a_{A\;,\;}A_{s}$, $b_{A}$ and σ. Thus, more parameters come into effect in the constitutive equation. As a result, the variability in these regions increases. The linear unloading curve also shows increased variability which continues from the austenite to martensite transformation region. At temperature −10 °C with maximum stress of 36 MPa, the material displays similar characteristics. At temperature −22.5 °C with maximum stresses of 21 MPa and 17 MPa, the linear loading region shows low variability while the phase transformation regions exhibits higher variability. At the linear loading region, only the parameter D is contributing, for which initial loading region shows low variability. In the transformation region, involvement of martensitic fraction integrating other parameters like $a_{M}$, $M_{s}$, T, $b_{M}$, $a_{A\;,\;}A_{s}$, $b_{A}$ and σ causes more parameters to come into effect in the constitutive equation (Equation (1)). Therefore, higher variability in these regions are prominent.

The sensitivity analysis of the Tanaka Model reveals that, when $T>A_{f}$, the model is sensitive to the elastic modulus D in the initial loading region. No other parameter shows influence in that region. In that region, martensitic fraction is zero and the analysis was done in isothermal temperature. So from Equation (1), it is clear that elastic modulus D is the only parameter for which the model is most sensitive. The parameter martensite start temperature $M_{s}$ shows low significance in the austenite to martensite phase transformation region. It comes into effect due to the fact that transformation of austenite to martensite starts at that region. In the loading phase transformation region, the model is sensitive to the phase transformation coefficient Ω. Martensitic fraction starts increasing at this zone from its zero value due to phase transformation. Therefore, phase transformation coefficient Ω is influential in this region. In the nonlinear unloading region, austenite start temperature $A_{s}$ becomes an influential parameter. In this region, martensite to austenite transformation starts, so the significance of $A_{s}$ is expected. 

For temperature region $A_{s}<T<A_{f}$, the sensitivity analysis of the Tanaka model shows that D is the most influential parameter in the initial loading region. But compared to $T>A_{f}$, the average sensitivity index of elastic modulus D is lower for $A_{s}<T<A_{f}$. This is because austenite to martensite transformation stress is higher for $T>A_{f}\;$than $A_{s}<T<A_{f}$. Also, the span of stress is higher for $T>A_{f}$. It can be observed from Figure 5a, for example, stress span is 0 to 40 MPa and then 40 MPa to 0. On the other hand, stress span is lower for Figure 5c. Phase transformation coefficient Ω is the second most influential parameter as per the average sensitivity index. Temperature T and martensite start temperature $M_{s}\;$show low significance for $A_{s}<T<A_{f}$ temperature region. Their Sobol total indices are greater than main indices which shows that interaction effects are higher for them than other parameters (Figure 6c,d). The total indices here refers to the main effect of the parameter as well as the interaction terms involved. Higher total indices thus signify higher interaction among the parameters including the main effect of a particular parameter. 

The Liang-Rogers model utilizes a cosine function for calculating martensitic transformation during the transformation regions. As a result, the stress-strain curve is convex-shaped for this model whereas it is concave-shaped for the Tanaka model. In the Liang-Rogers model, at temperature −10 °C with maximum stress of 40 MPa and 36 MPa, linear loading region shows low variability compared to the nonlinear loading region. The significant variability is seen in the phase transformation regions where austenite is transformed to martensite and vice versa. In the linear loading region, the material is initially at 100% austenite phase, i.e., no phase transformation is present in this region and the martensitic fraction is always zero. Therefore, T, θ and Ω were not contributing in the constitutive equations. In the phase transformation regions, martensite fraction comes into effect involving the cosine function in Equations (11) and (12). These equations involve the parameters$\;\xi_{A}$, $\xi_{M}$, $a_{M}$, $M_{f}$, T, $b_{M}$, $a_{A\;,\;}A_{s}$, $b_{A}$ and σ. Thus, more parameters come into effect in the constitutive equation (Equation (1)). As a result, the variability in these regions increases. 

At a temperature of −22.5 °C with maximum stresses of 21 MPa and 17 MPa, the Liang-Rogers model shows increased variability in the phase transformation regions than the initial loading region. This is because of the fact that martensitic fraction is zero in the initial loading region and the loading and unloading were done isothermally. As a result, as per Equation (1), only the elastic modulus D contributes to the output variability. This is the cause of low variability in the initial loading region. In the phase transformation regions, martensitic fraction can increase or decrease as prescribed by Equations (11) and (12). These equations involve the parameters $\xi_{A}$, $\xi_{M}$, $a_{M}$, $M_{f}$, T, $b_{M}$, $a_{A\;,\;}A_{s}$, $b_{A}$ and σ. Thus, more parameters come into effect in the constitutive equation (Equation (1)) including phase transformation coefficient Ω. As a result, variability in these regions increases. 

As per the sensitivity analysis for the Liang-Rogers model, for all four cases, the material is initially sensitive to elastic modulus D in the linear loading region. Then, upon further loading, the material enters into the phase transformation region where it transforms from austenite to martensite. From the sensitivity index distribution in Figure 8a, it can be seen that the martensite start and finish temperatures show some contribution to the model sensitivity. Equation (10) shows that martensitic fraction is a function of martensite finish temperature that causes $M_{f}$ to come into effect. Martensite start temperature $M_{s}$ shows contribution as austenite is being converted to martensite at that region. So, the temperature associated with martensite formation comes into effect. However, Figure 9a infers that the contribution of $M_{s}$ and $M_{f}$ are not significant. 

At the end of the loading and in the linear unloading region (Figure 8a), both elastic modulus D and phase transformation coefficient Ω show contribution to the model sensitivity. Also, $A_{s}$ and $A_{f}$ shows contribution to the model sensitivity during martensite to austenite phase transformation region. It is due to the fact that martensite is being converted to austenite in that region. However, it is seen that from the average sensitivity indices (Figure 9a), they are not significant through the total span of loading and unloading. From Sobol average sensitivity indices (Figure 9a,b) and eFAST average sensitivity indices (Figure 10a,b), it is observed that elastic modulus D is the most significant parameter. The second most influential parameter is the martensite finish temperature $M_{f}$. This is the case for temperature −10 °C ($T>A_{f}$).

For temperature −22.5 °C ($A_{s}<T<A_{f}$), it can be observed from Figure 9c that phase transformation coefficient Ω is the second influential parameter T and operating temperature is the third influential parameter. Both of them show some contribution. Figure 9d presents that elastic modulus D is the most influential parameter. Then operating temperature T is the second contributing parameter and martensite finish temperature $M_{f}$ is the third influential parameter. 

Based on the discussions above, the significant parameters have been listed in Table 5 for Tanaka and Liang-Rogers models. For engineering applications or further research utilizing these SMA models, it is recommended to observe the parameters listed here, and the associated uncertainty in them should be kept least in order to avoid failure or unbounded output.

Finally, the results obtained from Sobol sensitivity analysis for the Tanaka and Liang-Rogers models were verified using the extended FAST (eFAST) analysis, which can be observed from Figure 7 and Figure 10. They match closely, validating the Sobol sensitivity analysis for both models. 

## 6. Conclusions 

In this study, sensitivity and uncertainty analysis have been performed on two of the most widely used shape memory alloy constitutive models: the Tanaka and Liang-Rogers models. It was observed that any variability present in the input model parameters can have a significant impact on the output. The propagation of uncertainty has been presented at different operating temperatures and loading conditions. In order to determine which parameters have the most significance in the output variability, two different sensitivity analyses have been conducted. From these analyses, the most influential parameters for each model have been identified. The outcome of the study will help in designing real-life engineering applications by preventing failure which can be caused due to the uncertainty present in the design parameters. The models analyzed are for a particular material with certain loading and operating temperature conditions. This study can be extended by considering another SMA models or changing the material, loading conditions and the operating temperatures. 

## Figures and Tables

**Table 1:** Material properties for Cu-Zn-Sn [44].

Parameter	Description	Deterministic Value	Unit
T	Operating temperature	−10, −22.5	°C
D	Elastic modulus value	7 × 10^3^	MPa
Ω	Phase transformation coefficient	−7 × 10^1^	MPa
θ	Thermal coefficient of expansion	−7 × 10^−2^	MPa/°C
$M_{s}$	Martensite start temperature	−27	°C
$M_{f}$	Martensite finish temperature	−34	°C
$A_{s}$	Austenite start temperature	−25	°C
$A_{f}$	Austenite finish temperature	−14	°C

**Table 2:** Simulated operating temperature.

Temperature, T (°C)	Maximum Stress (MPa)	Region
−10	40, 36	$T>A_{f}$
−22.5	21, 17	$A_{s}<T<A_{f}$

**Table 3:** Probability distribution for input parameters.

Parameter	Distribution	Mean Value	Standard Deviation	Unit
T	Normal	−10, −22.5	0.1, 0.225	°C
D	Normal	7 × 10^3^	70	MPa
Ω	Normal	−70	0.7	MPa
θ	Normal	−7 × 10^−2^	7 × 10^−4^	MPa/°C
$M_{s}$	Normal	−27	0.27	°C
$M_{f}$	Normal	−34	0.34	°C
$A_{s}$	Normal	−25	0.25	°C
$A_{f}$	Normal	−14	0.14	°C

**Table 4:** Maximum strain variability.

Operating Temperature, T (°C)	Maximum Variability	
	Tanaka Model	Liang-Rogers Model
−10	56–137%	22–28%
−22.5	82–583%	48–105%

**Table 5:** Most influential parameters for Tanaka and Liang-Rogers models.

Temperature, T (°C)	Tanaka Model Parameters	Liang-Rogers Model Parameters	SMA Behavior
−10 ($T>A_{f}$)	D, $A_{s,\;}$$M_{s}$	D, $M_{f}$	Pseudoelastic Effect
−22.5 ($A_{s}<T<A_{f}$)	D, $\Omega ,\;T,\;M_{s}$	D, Ω,$\;T,M_{f}$	Shape Memory Effect
